# Surgeon workload and survival from breast cancer

**DOI:** 10.1038/sj.bjc.6601148

**Published:** 2003-07-29

**Authors:** J Stefoski Mikeljevic, R A Haward, C Johnston, R Sainsbury, D Forman

**Affiliations:** 1 Cancer Medicine Research Unit, Cancer Research UK, St James's Hospital, Leeds LS9 7TF, UK; 2 Northern and Yorkshire Cancer Registry and Information Services, Leeds LS16 6QB, UK; 3 Department of Surgery, Royal Free University College London, London W1W 7EJ, UK; 4 Department of Epidemiology and Health Services Research, University of Leeds, Leeds LS2 9JT, UK

**Keywords:** Breast neoplasms, workload, surgery, survival

## Abstract

The formation of multidisciplinary breast teams across the UK is intended to concentrate the assessment and treatment of breast cancer into the hands of high volume specialists. We undertook a retrospective population-based study in order to determine the trends in surgeon breast cancer workload in Yorkshire, UK, and to investigate whether patients treated by low-workload surgeons had poorer survival. Of 11 329 female breast cancer patients diagnosed in 1989–1994 in Yorkshire, 6% were managed by surgeons with a mean annual workload of less than 10 new patients, while surgeons with workloads of 10–29, 30–49 and >50 treated 21, 21 and 52%, respectively. Over the study period, increasing number of patients were managed by surgeons with higher workloads. Patients treated by low-workload surgeons had poorer survival. Five-year survival was 60% in the lowest workload category compared to 68% in the highest category. The relative risk of death was increased by 15% (RR=1.15, 95% CI 1.03–1.28) and by 10% (RR=1.10, 95% CI 1.02–1.18) for patients managed by surgeons with workloads <10 and 10–29 cases per annum in comparison to patients managed by surgeons with workloads of >50. The results of this study suggest increasing site specialisation in breast cancer among general surgeons. It also provides further evidence that the management of patients by surgeons with low workloads decreases overall survival.

Despite evidence of improving mortality from breast cancer in the UK (Peto *et al*, 2000), published studies suggest that survival from breast cancer in the UK compares unfavourably with many European countries (Quinn *et al*, 1998; Berrino *et al*, 1999). Wide variations in the management of breast cancer patients have been reported in the UK (Chouillet *et al*, 1994; Sainsbury *et al*, 1995b; Richards *et al*, 1996), and variable care is thought to contribute to the relatively poor performance in the UK (Sant *et al*, 1998). Greater specialisation in the management of breast cancer, with the formation of multidisciplinary teams, gradually followed the implementation of breast screening 10–12 years ago. This was further stimulated by a major national policy initiative in cancer in 1995 (Calman and Hine, 1995), and by the subsequent publication of the Improving Outcomes Guidance for breast cancer services in 1996 (Cancer Guidance, 1996).

Whether better outcomes in breast cancer are directly related to the concentration of breast cancer patients in the hands of specialist surgeons, and the multidisciplinary teams in which they work, has been a subject of growing interest. Although significant outcome benefits from higher caseloads for complex radical cancer surgery, such as in pancreatic or prostate cancer, have been reported, the evidence for caseload outcome relationships in cancers requiring low-risk surgery, like breast cancer, is based on far fewer studies (Hillner *et al*, 2000).

In this study, our aim was to follow-up an earlier investigation on the effect of surgeon workload on survival of breast cancer patients (Sainsbury *et al*, 1995a), and to establish whether the original finding, supportive of a volume outcome relationship, has remained the case in a more recent time period. We also wished to examine how surgeons' breast cancer workloads have changed over time and to assess whether differences in outcome could be explained by case-mix and treatment regimes employed.

## MATERIALS AND METHODS

All female breast cancer patients diagnosed in the former Yorkshire Regional Health Authority area between 1989 and 1994 were identified from the Northern and Yorkshire Cancer Registry and Information Service (NYCRIS) database (*n*=12, 338). Patients treated outside the region, those registered on the basis of information available from death certificates only, and some private patients were excluded because of the insufficient clinical management details (*n*=520). The total number of patients eligible for the study was 11 818.

Relevant patient characteristics and treatment details were abstracted from the cancer registry database. Socioeconomic status was determined using small-area data on socioeconomic classification from Super Profiles, a tool created by clustering together socially similar enumeration districts (Aveyard *et al*, 2000). The surgeon workloads were calculated as the mean number of new breast cancer patients managed per practising year. Patients were divided into four categories according to their surgeon annual workload (very low workload category of <10, low category 10–29, moderate 30–49 and high workload category with >50 average annual new cases). The mean annual hospital workload categories were estimated taking into account new consultant posts and retirements during the study period.

### Data analysis

The survival period for each patient was calculated as the time difference between the date of diagnosis (taken as date of first hospital visit) and the date of death or censoring (1 January 2002). The overall relative survival and the relative survival for each factor (disease extent, treatment modality, type of surgery, socioeconomic status, time period, surgeon and hospital workload) were estimated using the Kaplan–Meier method. Cox's proportional hazards model was applied to estimate the relative risk of death (Cox, 1972). Estimates were initially considered for each factor in isolation, and then all case-mix factors (age, disease extent, tumour grade, socioeconomic status and time period) were included in the regression model. Finally, the relative risks of death were adjusted for all factors together, including treatment, surgeon workload and hospital volume.

We have compared our findings with the results of an earlier study that investigated the influence of surgeon workload on survival from breast cancer in Yorkshire for the period 1979–1988 (Sainsbury *et al*, 1995a). That study was based on data from the same cancer registry and covered the population of the same region (the former Yorkshire Regional Health Authority area). The same workload categories have been used, and the same statistical methods applied. We used the original data from the earlier study to re-estimate the relative risks of death with the high-workload group as a base category in order to show the disadvantage associated with being managed by low-workload surgeon.

## RESULTS

Over the study period, 95 surgeons were involved in the management of 96% of the study population (*n*=11 329), either with or without additional involvement of a consultant from another specialty. One-third (36.9%) of all cases were managed solely by a surgeon.

Overall, surgeons with a mean annual workload of less than 10 managed just 6% (*n*=702) of the study population (Table 1

**Table 1:** Number (percentage) of patients in each workload category by time period

**Surgeon workload**	**1989–1990**	**1991–1992**	**1993–1994**	**Overall**
<10	359 (10.0%)	199 (5.1%)	144 (3.7%)	702 (6.2%)
10–29	882 (24.5%)	790 (20.5%)	677 (17.5%)	2349 (20.7%)
30–49	756 (21.0%)	774 (20.1%)	830 (21.4%)	2360 (20.8%)
50+	1602 (44.5%)	2091 (54.3%)	2225 (57.4%)	5918 (52.2%)
Total	3599 (31.8%)	3854 (34.0%)	3876 (34.2%)	11329 (100%)

). Surgeons with a workload of 10–29 and 30–49 treated the same proportion of cases (21%), while the high-workload surgeons managed 52%. The proportion of patients treated by surgeons with workload of more than 30 has increased steadily during the study period from 66% in 1989–1990 to 79% in 1993–1994 (Table 1), what is a substantial increase over the 45% during the early and mid-1980s (Table 2

**Table 2:** Proportion of patients and consultants in each workload category and results of the adjusted relative risks of death compared with the earlier study

	**% patients**		**% surgeons**		**Adjusted relative risk (95% confidence interval)**	
**Workload category**	**1979–1988^a^**	**1989–1994**	**1979–1988^a^**	**1989–1994**	**1979–1988^b^**	**1989–1994**
<10	10%	6%	67%	49%	1.16 (1.06–1.27)	1.15 (1.03–1.28)
10–29	45%	21%	24%	27%	1.14 (1.07–1.21)	1.10 (1.02–1.18)
30–49	15%	21%	5%	12%	0.98 (0.90–1.05)	1.01 (0.93–1.08)
50+	30%	52%	4%	12%	1.00	1.00

a Source: Sainsbury *et al* (1995).

b Figures based on the above paper (see Materials and methods section).

). The overall number of surgeons that were treating breast cancer patients in the region fell from 180 in 1979–1988 to 95 in 1989–1994. In 1989–1994, 47 (49%) of the 95 surgeons were in the <10 category, compared with 67% in 1979–1988. A total of 11 surgeons were treating >50 patients per year in 1989–1994.

There were no significant differences in the type of surgery (mastectomy *vs* breast-conserving surgery) by workload category (Table 3

**Table 3:** Number (percentage) of patients in each workload category by type of surgery and adjuvant therapy received

	**Workload**				
	**<10**	**10–29**	**30–49**	**50+**	**All**
*Surgery type*					
BCS alone	294 (41.9%)	757 (32.2%)	958 (40.6%)	2178 (36.8%)	4187 (37.0%)
Mastectomy alone	246 (35.0%)	971 (41.3%)	743 (31.5%)	2272 (38.4%)	4232 (37.4%)
BCS & M	75 (10.7%)	332 (14.1%)	332 (14.1%)	854 (14.4%)	1593 (14.1%)
					
*Treatment modalities received in addition to surgery*					
Radiotherapy	298 (42.5%)	1112 (47.3%)	1207 (51.1%)	2769 (46.8%)	5386 (47.5%)
Chemotherapy	85 (12.1%)	255 ((10.9%)	397 (16.8%)	1226 (20.7%)	1963 (17.3%)
Hormone therapy	498 (70.9%)	1606 (68.4%)	1606 (68.1%)	4256 (71.9%)	7966 (70.3%)
RT+CT/HT	268 (38.2%)	944 (40.2%)	1083 (45.9%)	2536 (42.9%)	4831 (42.6%)
Surgery alone	71 (10.1%)	218 (9.3%)	218 (9.2%)	481 (8.1%)	988 (8.7%)

BCS=breast-conserving surgery; M=mastectomy; S=surgery; CT=chemotherapy; RT=radiotherapy and HT=hormone therapy.

). The use of chemotherapy was the only treatment modality strongly correlated with consultant workload. Chemotherapy rates increased from 12% in the very low workload category to 21% in the high category. Patients managed by surgeons with an annual workload over 30 were less likely to be treated by surgery alone and more likely to receive multimodality primary treatment (surgery combined with radiotherapy, chemotherapy and/or hormone therapy) (Table 3).

There were large differences between hospital trusts in the proportions of patients managed by surgeons in each workload category. Surgeons in the highest workload category (>50 cases per annum) managed nearly all patients (98.8%) in one trust in contrast to six other hospital trusts (out of 17 in total) where no surgeons worked at that caseload level.

The overall survival 5 years after diagnosis was 66%. Patients treated by higher workload surgeons had better survival. The 5-year survival was 60% in the lowest surgeon workload category, 64 and 66% in the intermediate categories and 68% in the highest workload category. Multivariate analysis confirmed the survival advantage for patients treated by moderate- and high-workload surgeons. Compared to a baseline of 1.00 for patients treated by surgeons with the high workload, the relative risk of death increased to 1.01, 1.10 and 1.15 in each subsequent category after adjusting for all recorded case-mix factors and treatment (Table 4

**Table 4:** Relative risk of death (95% confidence intervals)

		**Relative risk ratios**					
		**Each factor alone**		**Allowing for case-mix factors**		**Allowing for all factors**	
*Case-mix*							
Age (years)	<50	1.00		1.00		1.00	
	50–64	1.06	(0.97–1.15)	1.12	(1.03–1.22)	1.25	(1.14–1.36)
	65–74	1.86	(1.70–2.02)	1.88	(1.73–2.05)	2.01	(1.82–2.22)
	75+	3.64	(3.35–3.95)	3.71	(3.41–4.02)	3.24	(2.92–3.60)
Extent of disease	No known nodes/metastases Nodal involvement	1.00		1.00		1.00	
		1.67	(1.58–1.77)	1.77	(1.67–1.88)	1.80	(1.70–1.92)
	Metastases	7.81	(7.13–8.55)	6.58	(6.00–7.20)	5.16	(4.67–5.69)
Tumour grade (differentiation)	Well	1.00		1.00		1.00	
	Moderately	1.51	(1.32–1.73)	1.40	(1.22–1.61)	1.42	(1.23–1.63)
	Poor	2.53	(2.22–2.87)	2.34	(2.06–2.66)	2.21	(1.94–2.52)
	Not known	2.35	(2.07–2.67)	2.09	(1.84–2.38)	1.65	(1.45–1.88)
Socioeconomic status	1–3 (affluent)	1.00		1.00		1.00	
	4–7	1.17	(1.09–1.25)	1.10	(1.03–1.17)	1.07	(1.00–1.15)
	8–10 (deprived)	1.32	(1.22–1.43)	1.26	(1.16–1.36)	1.22	(1.13–1.33)
Time period	1989–1990	1.00		1.00		1.00	
	1991–1992	0.98	(0.93–1.04)	0.93	(0.87–0.99)	0.92	(0.86–0.98)
	1993–1994	0.85	(0.80–0.90)	0.93	(0.87–1.00)	0.88	(0.82–0.94)
*Treatment*							
Treatment	S+R+C+H	1.00		1.00		1.00	
	S+R+C	1.14	(0.95–1.37)	1.17	(0.98–1.41)	1.17	(0.98–1.41)
	S+R+H	0.89	(0.79–0.99)	0.76	(0.68–0.86)	0.75	(0.67–0.85)
	S+R	0.72	(0.61–0.86)	0.81	(0.68–0.96)	0.79	(0.67–0.95)
	S+C+H	1.12	(0.94–1.32)	1.13	(0.95–1.34)	1.14	(0.96–1.35)
	S+C	1.01	(0.78–1.30)	1.02	(0.79–1.32)	1.03	(0.80–1.32)
	S+H	1.05	(0.94–1.17)	0.80	(0.70–0.90)	0.79	(0.70–0.89)
	S	0.80	(0.69–0.92)	0.77	(0.67–0.90)	0.78	(0.67–0.90)
	R+C+H	6.07	(4.50–8.19)	2.69	(1.98–3.66)	2.67	(1.96–3.63)
	R+C	9.25	(5.21–16.42)	6.58	(3.69–11.72)	6.46	(3.62–11.50)
	R+H	4.66	(3.84–5.65)	1.49	(1.20–1.83)	1.47	(1.19–1.82)
	R	1.56	(0.86–2.83)	1.01	(0.55–1.84)	1.00	(0.55–1.82)
	C+H	8.46	(6.35–11.27)	3.52	(2.62–4.72)	3.54	(2.64–4.76)
	C	2.98	(1.41–6.29)	1.75	(0.83–3.70)	1.75	(0.83–3.70)
	H	4.53	(4.02–5.10)	2.18	(1.89–2.52)	2.19	(1.90–2.53)
	None	8.42	(6.49–10.92)	5.22	(3.98–6.86)	5.28	(4.02–6.94)
							
*Workload*							
Surgeon Workload (cases per year)	50+	1.00		1.00		1.00	
	30–49	1.08	(1.01–1.16)	1.06	(0.99–1.14)	1.01	(0.93–1.08)
	10–29	1.20	(1.12–1.28)	1.05	(0.98–1.13)	1.10	(1.02–1.18)
	<10	1.27	(1.14–1.41)	1.10	(0.99–1.23)	1.15	(1.03–1.28)
Hospital Workload (cases per year)	100+	1.00		1.00		1.00	
	70–99	1.01	(0.94–1.08)	1.00	(0.93–1.07)	1.02	(0.95–1.10)
	50–69	1.05	(0.97–1.13)	1.02	(0.95–1.10)	1.06	(0.97–1.14)
	<50	0.94	(0.86–1.01)	0.99	(0.91–1.08)	0.95	(0.87–1.03)

S=surgery; CT=chemotherapy; RT=radiotherapy and HT=hormone therapy.

). For the workload categories 10–29 and less than 10, the relative risks of death were similar to those described in the earlier paper (Table 2). The effect of surgeon workload on survival contrasted with the absence of any significant hospital workload effect.

Patient age significantly influenced survival, with the relative risk of death increasing with increasing age (Table 4). Patients with more advanced disease (positive axillary lymph nodes or metastases) and less-well-differentiated tumours had poorer survival. Socioeconomic status also influenced the survival of breast cancer patients with the least advantaged group having a significantly increased risk of death (1.22 (95% CI 1.13–1.33)) compared to the most advantaged. Survival improved gradually over the study period. Compared to the baseline of 1.00 for patients treated in 1989 and 1990, relative risk reduced to 0.92 (0.86–0.98) for those treated between 1991 and 1992, and down to 0.88 (0.82–0.94) in the most recent period (1993–1994).

All treatment combinations in which surgery was not performed did poorly, while most combinations of treatment that included surgery were not statistically significant from the base category. The only exception was the observation that patients who had surgery and adjuvant radiotherapy and/or hormone therapy had a lower relative risk than those in whom chemotherapy was given.

## DISCUSSION

This study provides further evidence to support the hypothesis that management by high-workload surgeons improves overall survival from breast cancer. Patients managed by surgeons with workloads less than 30 new cases per year had a 4% lower survival at 5 years and a 10% increase in the relative risk of death in comparison to management by high-workload surgeons (more than 50 new cases year^−1^). This is consistent with the previously described findings for the 1979–1988 time period (Sainsbury *et al*, 1995a). The survival disadvantage could not be explained by any differences in the case-mix or treatment patterns in both studies and has remained consistent across this population for 16 years. During that period (1979–1994), there was a significant increase in the proportion of patients treated by high-workload surgeons and improvement of the overall 5-year survival from 63% (YCO, 1995) to 66%.

The current UK policy recommendation for specialist breast cancer teams is that every team should see at least 100 new cases per year (Calman and Hine, 1995). This guidance supports surgical specialisation and team working but does not define a lower threshold for new breast cancer cases per individual clinician. This may be attributable to the scarcity of evidence on the relationship between surgical caseloads and outcomes for this disease. There is evidence available from other large observational studies supporting both hospital caseload (Roohan *et al*, 1998) and surgical specialisation (Gillis and Hole, 1996) as important determinants of better survival in relation to breast cancer.

This was an observational population-based study of all breast cancer cases diagnosed within a defined geographical area covered by the regional cancer registry. One advantage of large-scale observational methods is that potential biases that may operate in small studies are minimised when large numbers of cases are examined on a population basis (Haward and Forman, 1996). More accurate and unbiased assessment of the relationship between volume and outcome could be obtained if the data could be adjusted for other important case-mix variables that influence outcomes (Devenport *et al*, 1996). In this study, we included all breast cancer cases diagnosed within a population of over 3.6 million, and estimated the relative risk of death in relation to surgical workload after adjusting for a range of patient factors of established importance. While these factors were less comprehensive than the ideal, particularly the completeness of the assessment of extent of disease, there is no reason to believe that these weaknesses could bias such a large population sample in relation to surgeon caseloads. For this to occur would require primary care physicians to selectively refer more severe breast cancer cases (i.e., those with worse prognostic features) to lower caseload surgeons. Given that primary care physicians generally refer only suspected cases (without the benefit of detailed mammographic assessment and histology) for diagnosis and assessment, with a ratio of at least 10 without malignant disease for every cancer diagnosed, such systematic bias seems implausible. The converse, whereby potentially more serious cases were referred to specialists seems more probable and, if this factor could be adjusted for, would increase the magnitude of our observed effect.

The proportion of cases treated in Yorkshire by surgeons who managed more than 30 new breast cancer patients per year increased from 45% in 1979–1988 to 73% in 1989–1994. Increased breast cancer incidence coupled with more subspecialisation among general surgeons are the most likely causes for this increase. The increasing proportion of patients managed by surgeons with high breast workloads suggests the trend towards subspecialisation among surgeons began before this became an explicit recommendation following the Calman–Hine policy initiative. However, this early trend was not consistent within the Yorkshire region where large differences were found in the proportions of patients managed by surgeons in each workload category according to the NHS trust where they received their care.

It was not possible from our data to determine whether high-workload surgeons have better outcomes because large caseloads enable them to enhance particular surgical skills or because larger volumes increase their expertise in breast cancer management, using all treatment modalities more appropriately. However, our data showed that patients managed by surgeons with higher workloads received proportionally more chemotherapy and were more likely to receive the combination of radiotherapy and other adjuvant therapies. Chemotherapy and hormone therapy have been repeatedly demonstrated to reduce the risk of local recurrence (EBCTCG, 1992) and improve survival in appropriate patients (EBCTCG, 1998; Aapro, 2001), although the overall use of chemotherapy during the study period was low. The information on local recurrence was not available in this study but there is evidence that specialisation reduces the risk of local recurrence due to increased use of systemic therapy (Golledge *et al*, 2000). The negative impact of chemotherapy in the multivariate analysis was most likely to have been due to the selection of the poorest prognosis individual patients for this treatment during the period in question. This predates the more general application of chemotherapy protocols to groups of patients at higher risk, which are now in use. The increased use of combined adjuvant therapy by high-workload surgeons strongly suggests a multidisciplinary approach and input in the management of their breast cancer patients. In contrast, low-workload surgeons were likely to neither interact with medical and clinical oncologists nor attend multidisciplinary meetings. It is also probable (although we have no data to confirm this) that their surgical skills in achieving complete excision were not as good as the experienced surgeon. There are data from Edinburgh indicating that experienced surgeons achieved higher rates of complete excision and had lower mastectomy rates for cases of DCIS (Mr M Dixon, Western General Hospital, personal communication).

During the 16-year period, surgeons with high workload treated progressively more breast cancer patients and there was an improvement in survival rates over the same period. Although a number of factors are likely to have contributed to this improved survival including advances in diagnosis and treatment as well as better organisation of care, we confirmed that surgeon workload does have a significant effect. These results support our earlier findings, and provide further evidence of the benefits that follow greater surgical specialisation within the context of moves towards more multidisciplinary organisation of breast cancer services.
